# The *STK11* gene F354L mutation is associated with male spermatogenic dysfunction

**DOI:** 10.1038/s41598-026-53888-4

**Published:** 2026-05-25

**Authors:** Neng Wan, Xiang Huang, Gang Jing, Laifeng Ren

**Affiliations:** 1 Central Laboratory, Shanxi Province Cancer Hospital/Shanxi Hospital Affiliated to Cancer Hospital, Chinese Academy of Medical Sciences/Cancer Hospital Affiliated to Shanxi Medical University, Taiyuan, 030001 Shanxi China; 2 Reproductive Medicine Center of Shanxi Maternal and Child Health Hospital, No. 13 Xinmin North Street, XinghualingDistrict, Taiyuan, 030001 Shanxi People’s Republic of China; 3 The Clinical Laboratory, The Second Hospital of Shanxi Medical University, The Second Hospital of Shanxi Medical University, No. 382, Wuyi Road, Taiyuan, 030001 Shanxi People’s Republic of China

**Keywords:** Genetics, Oncology, Pathogenesis, Urology

## Abstract

To investigate the probability of the *STK11* gene F354L mutation in normal individuals and infertile males with spermatogenic dysfunction, and to predict its impact on male spermatogenic function. We analyzed the F354L coding sequence in 192 azoospermia and severe oligozoospermia patients (patient group) and 199 normal fertile males (control group) using PCR and DNA sequencing. We detected 16 cases of the mutation in the patient group, but 7 cases were also found in the control group. The mutation rate showed some significant statistical difference between the two groups (*p* = 0.043). Immunofluorescence staining revealed that STK11 is highly expressed in mouse testicular tissues and localized to the midpiece of human and mouse sperm. Lentivirus-mediated overexpression of wild-type and F354L-mutant STK11 in TCAM2 cells demonstrated that the F354L variant reduces AMPK phosphorylation and disrupts cellular polarity, as evidenced by Western blot and Golgi reorientation assays. These findings suggest that the F354L mutation may impair spermatogenesis and sperm motility by perturbing metabolic homeostasis and cell polarity.

**Supplementary Information:**

The online version contains supplementary material available at 10.1038/s41598-026-53888-4.

## Introduction

Male infertility is a major cause of reproductive failure and affects a large number of families worldwide^1^. In recent years, genetic mutations, as one of the significant factors influencing male reproductive health, have become a research focus^2^.

The *STK11* gene on human chromosome 19 encodes an important serine/threonine kinase. This protein, also known as LKB1, is 433 amino acids in length and is encoded by nine exons.STK11 is an upstream tumor purifier and kinase that regulates various cellular functions, including cell cycle, apoptosis, cell polarity and metabolism by activating AMPK and several AMPK-associated kinases^3,4^.The protein features a nuclear localization signal in its N-terminal region and a non-catalytic C-terminal domain. Mutations in STK11 have been linked to Peutz–Jeghers syndrome (PJS) and various malignancies, such as colon cancer, lung adenocarcinoma and liver cancer^5^.Notably, missense mutations within the C-terminal region of STK11—including P324L, F354L, and T367M—have been shown to disrupt both AMPK signaling and polarity functions^6^.

Several studies have suggested that mutations in the *STK11* gene play a crucial role in male spermatogenic dysfunction^7^. It has also been reported that loss of STK11 function disrupts cellular energy metabolism and ATP supply within spermatogenic cells, impairing their normal development^8^. Spermatogenesis is a complex process that requires a large amount of energy. Abnormalities in energy metabolism can affect the division and differentiation of spermatogenic cells^9^, thereby resulting in spermatogenic dysfunction.

Furthermore, *STK11* mutations may disturb cell cycle regulation in spermatogenic cells, leading to arrest at specific stages and compromising sperm production^10^. STK11 is also involved in the regulation of cell polarity and cytoskeletal organization. Mutations in the *STK11* gene can lead to abnormal changes in the structure and function of the cytoskeleton in spermatogenic cells, affecting the movement and shaping of sperm^11^.

Men with Peutz-Jeghers syndrome (PJS) often experience impaired spermatogenesis and have an elevated risk of Sertoli cell tumors^12^. Although *STK11* mutations are known to contribute to male infertility, the specific impact of the F354L variant on spermatogenesis remains poorly understood. In our preliminary study, whole-exome sequencing of peripheral blood from 114 patients with spermatogenic dysfunction (azoospermia or severe oligozoospermia) revealed 11 cases carrying the germline mutation *STK11* F354L, with no other pathogenic mutations detected in this gene. Therefore, we focused on *STK11* F354L as a primary research target to investigate its potential association with male reproductive function.

We further analyzed the F354L mutation frequency in a cohort of 192 infertile patients and 199 healthy male controls undergoing physical examination, which showed a significantly higher prevalence of the mutation in the patient group. Analysis of the Human Protein Atlas (HPA) single-cell RNA expression clusters indicated predominant expression of STK11 in spermatids, a finding corroborated by immunofluorescence staining in mouse testis sections. Moreover, staining of human and mouse sperm localized STK11 predominantly to the midpiece of the sperm flagellum. Finally, in vitro cellular assays demonstrated that the F354L mutation downregulates AMPK pathway activity and disrupts cell polarity. Our results indicate that the F354L mutation is more prevalent among infertile men and may disrupt spermatogenesis through mechanisms involving AMPK signaling and cell polarity.

## Methods

### Study subjects

This study included 391 male participants from the center of reproductive medicine, consisting of 192 patients with azoospermia or severe oligozoospermia (patient group) and 199 healthy male controls undergoing physical examination (control group). The patient group (*n* = 192) was defined by: sperm concentration < 15 × 10⁶/mL combined with total motility < 32%. The control group (*n* = 199) had parameters exceeding both thresholds. All subjects underwent detailed clinical examinations, including reproductive history, physical examination, sperm quality (two analyses at least), testicular size (determined by ultrasound), exclusion of known genetic abnormalities (chromosome karyotype, Y chromosome microdeletions), endocrine disorders (sex hormone tests), and environmental factors that may interfere with spermatogenic function.

### Semen analysis

Semen analysis is mainly based on the standards set by the World Health Organization. (2010) WHO Laboratory Manual for the Examination and Processing of Human Semen, 5th edition. World Health Organization, New York. Routine analysis requires the sample to be collected within 2 to 7 days after the sexual abstinence. The analysis should be performed three times, with each test interval being more than 3 weeks.

### Sex hormone testing

Sex hormones were detected using the chemiluminescence method from Beckman Coulter. Both intra-group and inter-group variations were less than 10%. The normal range of serum FSH, LH and testosterone level was 2.1–18.6 IU/L, 1.7–11.2 IU/L and 262–870 ng/dl.

### DNA extraction

Clinical blood samples from both patient and control groups were processed as follows:200 µL whole blood was mixed with 2 mL 1× hemolytic agent (Beijing TongshengShidai Biotech, Cat. 20231120), vortexed for 30 s, and incubated for 20 min at room temperature. Lysates were centrifuged at 3,000 × g for 5 min. The supernatant was discarded, retaining the white membranous pellet. DNA was extracted from pellets using the TIANamp Genomic DNA Kit (Tiangen Biotech, Cat. DP304) according to manufacturer’s protocol. Purified DNA was eluted in 55 µL buffer and transferred to 1.5 mL microcentrifuge tubes. DNA concentration was measured using a QuickDrop Spectrophotometer (Molecular Devices, USA).

### PCR amplification, agarose gel electrophoresis, and sanger sequencing

The DNA fragment of STK11 F354L locus was amplified by polymerase chain reaction (PCR) using genomic DNA extracted from peripheral venous blood as template, with forward primer S11-F (5’−3’: TGCTCCTAGAGGACATGGCT) and reverse primer S11-R (5’−3’: GCAGAAGCTGTCCTTGTTGC). PCR conditions were: initial denaturation at 95 °C for 5 min; 40 cycles of denaturation at 94 °C for 30 s, annealing at 60 °C for 30 s, and extension at 72 °C for 1 min; and final extension at 72 °C for 5 min, yielding a 455-bp target fragment.

Amplified products were analyzed by 1% agarose gel electrophoresis, and samples were loaded as follows: Lane 1, 5µL 250-bp DNA ladder (Real-time, cat: RTM405, Lot:6608674); Lanes 2-N, 2 µL DNA samples. DNA fragments were visualized under UV illumination using a transilluminator (BLT GelView 6000 M). Positive amplicons were further analyzed by Sanger sequencing.

### Cell culture

The human testicular seminoma cell line TCAM-2 cells, purchased from Otwo Biotech (ShenZhen) Inc., were cultured in DMEM High Glucose Medium (BOSTER, China) supplemented with 10% fetal bovine serum (Every Green, China) and 1% penicillin/streptomycin (Solarbio) at 37 °C in a 5% CO₂ atmosphere.

### Lentivirus packaging and cell infection

Lentiviral vectors overexpressing human *STK11* wild-type or F354L mutant, as well as negative control lentivirus (empty vector), were purchased from GenePharma (Shanghai, China). TCAM-2 cells were seeded in 24-well plates at a density of 1 × 10⁵ cells/well and infected when cell confluence reached 60%. Stable cell lines were selected with 2 µg/ml puromycin (Invitrogen) at 48 h post-infection.

### Immunofluorescence assay

The cell samples or mouse/human sperm were fixed with 4% paraformaldehyde (PFA) for 10 min, followed by three washes with PBS. Samples were permeabilized with 0.3% Triton X-100 (BBI Life Science) for 10 min, then washed three times with PBS and blocked with blocking solution (2% BSA, 5% goat serum, 0.5% Triton X-100 in PBS) for 45 min. After blocking, samples were incubated with primary antibodies against STK11 (ABclonal, catalog No.: A22636; rabbit; 1:200 for IF) or GM130 (ABclonal, catalog No.: A11408; rabbit; 1:1,000 for IF) at 37 °C for 30 min, then with CY3-labeled secondary antibodies (Sigma, No.:C2306,1:200) at 37 °C for 30 min. Following three additional washes with PBS, samples were incubated with DAPI (Vector Laboratories) for nuclear counterstaining. Images were captured and analyzed using an Olympus fluorescence microscope (BX51).

### Western blotting analysis

Cells were washed twice with 1× PBS (Boster, China) and lysed in RIPA lysis buffer (Beyotime, China) supplemented with protease and phosphatase inhibitors (Roche, USA). After sonication on ice, lysates were centrifuged at 12,000×g for 10 min at 4 °C, and supernatants were collected. Protein concentrations were determined using a BCA assay (Boster, China). Equal amounts of protein (20 µg per lane) were boiled for 7 min in 1× SDS buffer (Boster, China), separated by 4%−20% SDS-PAGE (GenScript, USA), and transferred to 0.22 μm PVDF membranes (Millipore, USA). Membranes were blocked with 5% non-fat milk in 1× Tris-buffered saline with 0.1% Tween-20 (TBST) for 90 min at room temperature, then incubated overnight at 4 °C with gentle rocking in blocking buffer containing primary antibodies: STK11 (ABclonal, catalog No.: A22636; rabbit; 1:2,000 for WB), P-AMPK(T172) (CST, catalog No.: 3, 50081 S; rabbit; 1:1,000 for WB), Vimentin (Thermo, catalog No.: SD2369201L; rabbit; 1:10,000 for WB), GAPDH (Proteintech, catalog No.: HRP-60004; mouse; 1:10,000 for WB), and E-cadherin (CDH1; Boster, catalog No.: BST17884166; rabbit; 1:1,000 for WB). The following day, membranes were incubated with HRP-conjugated secondary antibodies (1:2,000; Sigma) for 1.5 h at room temperature. After three washes with TBST, protein bands were visualized using ECL chemiluminescence substrate (Millipore, USA) and imaged with a GelView 6000 M system (BLT, China). Densitometric analysis was performed using Fiji (Fiji Is Just ImageJ) version 2.14.0/1.54f (https://fiji.sc/), with relative protein expression normalized to GAPDH. Experiments were repeated three times.

### Golgi reorientation polarity assays

Following successful lentiviral transduction of TCAM-2 cells with *STK11*-F354L, *STK11*-WT, or empty vector controls, stable lines were established. At 80% confluency, scratch wounds were generated using sterile 10-µL pipette tips. Cells were fixed with 4% paraformaldehyde (PFA) at 0 and 9 h post-scratching. For Golgi positioning analysis, cells were immunostained for GM130 (Golgi marker) and DAPI (nuclear counterstain) as previously described. Cells with Golgi apparatus oriented toward the wound front were scored polarity-positive. Only the first row of cells adjacent to wound edges was quantified per replicate, with ≥ 300 cells analyzed per time point.

### Statistical analysis

Data were analyzed using IBM SPSS Statistics version 20.0 (https://www.ibm.com/products/spss-statistics). The comparison of F354L mutation frequencies between the two groups was performed using the Chi-square test (or Fisher’s exact probability test). Parameters of basic clinical characteristics of the participants included in the mutation screening was analyzed using t-tests and nonparametric test was used to evaluate indicators for non-normal distribution such as sperm concentration and sperm progressive motility rate (PR). *P* < 0.05 was considered statistically significant. Differences between various groups were detected using the one-way ANOVA, followed by the Dunnett’s multiple-range test for multiple comparisons using GraphPad Prism version 9.5.0 (GraphPad Software, USA). Data are expressed as mean ± SD and unpaired t test (*P < 0.05, **P < 0.01, ***P < 0.001, ****P < 0.0001, ns, no statistical significance).

## Results

### Clinical characteristics analysis

We compared clinical parameters between patient group and control group. Significant differences were found in volume of testes, sex hormones (FSH, LH), concentration of sperm and PR (*p* < 0.05). However, no significant differences were observed between patient group and control group concerning age, sex hormone(T). The results are shown as Table 1.

**Table 1:** Basic characteristics of the participants included in the mutation screening.

Parameters	Patient group(*n* = 192)	Control group(*n* = 199)	*P*
**Age**	31.59 ± 3.02	32.63 ± 3.31	0.873
**Volume of testes**	7.54 ± 2.81	13.89 ± 3.96	0.039
**FSH**	18.66 ± 5.13	4.27 ± 2.43	<0.001
**LH**	3.52 ± 0.37	1.13 ± 0.25	0.041
**T**	330.63 ± 38.58	450.21 ± 40.67	0.279
**Concentration of sperm (x10** ^**6**^ **)**	2.14(0.00–5.07.00.07)	65.33(20.96–197.87.96.87)	<0.001
**PR (%)**	6.81(0.00–9.94.00.94) ^A^	40.05(32.00–98.81.00.81)	<0.001

Note: The results are expressed as the mean ± SD or as median and range.

A: Only 123 patients were included (other 69 patients had sperm concentration less than 2 × 10^6^/ml and motility of which not applicable).

### The amplification and sequencing of the target gene *STK11*

**Fig. 1:**
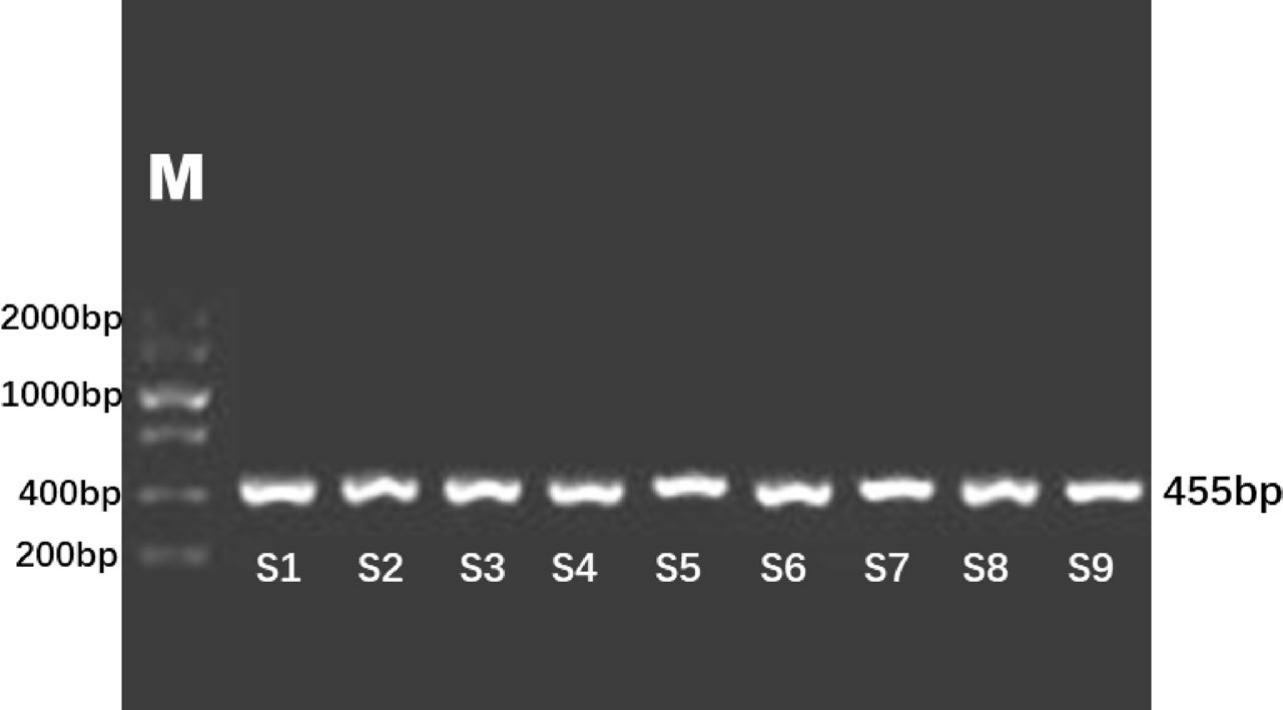
DNA gel electrophoresis of PCR-amplified *STK11* F354L fragments. A 455-bp target band is visible in clinical sample lanes. Lane M: RTM405 DNA Marker (200–2000 bp); Lanes 1–9 (S1-S9): PCR products from patient genomic DNA.

**Fig. 2:**
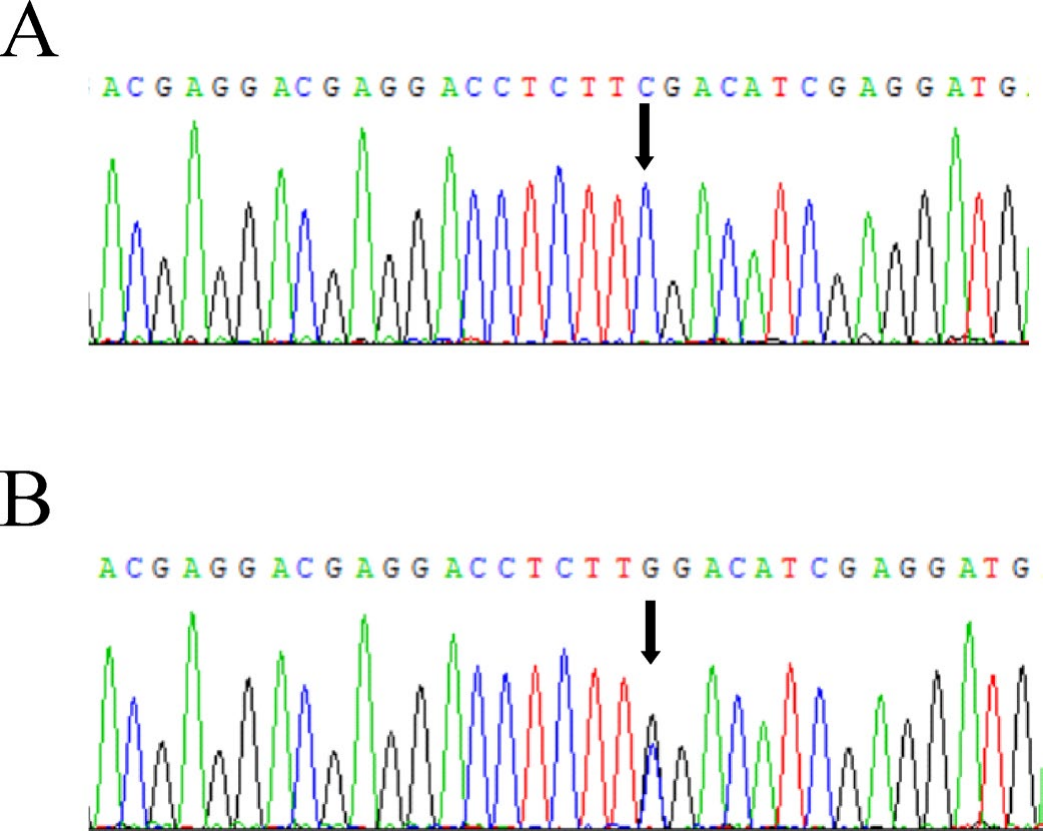
Sanger sequencing chromatograms of the *STK11* F354L locus. **(A)** Wild-type sequence. **(B)** Heterozygous missense mutation c.1062 C > G (p. Phe354Leu).

The DNA extracted from peripheral blood was amplified to obtain the target fragment through a PCR reaction, and then verified by DNA gel electrophoresis (Fig. 1). And the *STK11* F354L mutation was detected by Sanger sequencing(Fig. 2).

### *STK11* F354L mutation frequency analysis

DNA sequencing revealed that 16 cases of the F354L mutation occurred in the patient group, while 7 cases were found in the control group. Data were analyzed using SPSS 20.0 statistical software. The two groups were performed using the Chi-square test. The mutation frequency did show significant differences between the two groups (χ^2^ = 4.093, *p* = 0.043). The mutation frequencies in the patient and control groups were 8.33% and 3.52%, respectively. All results are shown in Table 2. Further analysis indicates that, among the 16 patients carrying the F354L mutation, only 9 underwent assisted reproductive technology. Their embryo quality did not show a significant difference when compared to other non-mutated patients in the same group (data not shown).

**Table 2:** The comparison of F354L mutation frequencies between the two groups.

Group	Positive	Negative	Total	Mutation Frequency/%	χ²	*p*
**Patient group**	16	176	192	8.33		
**Control group**	7	192	199	3.52		
**Total**	23	368	391	5.88	4.093	0.043

### STK11 is highly enriched in spermatids and localized to the midpiece of mature sperm

**Fig. 3:**
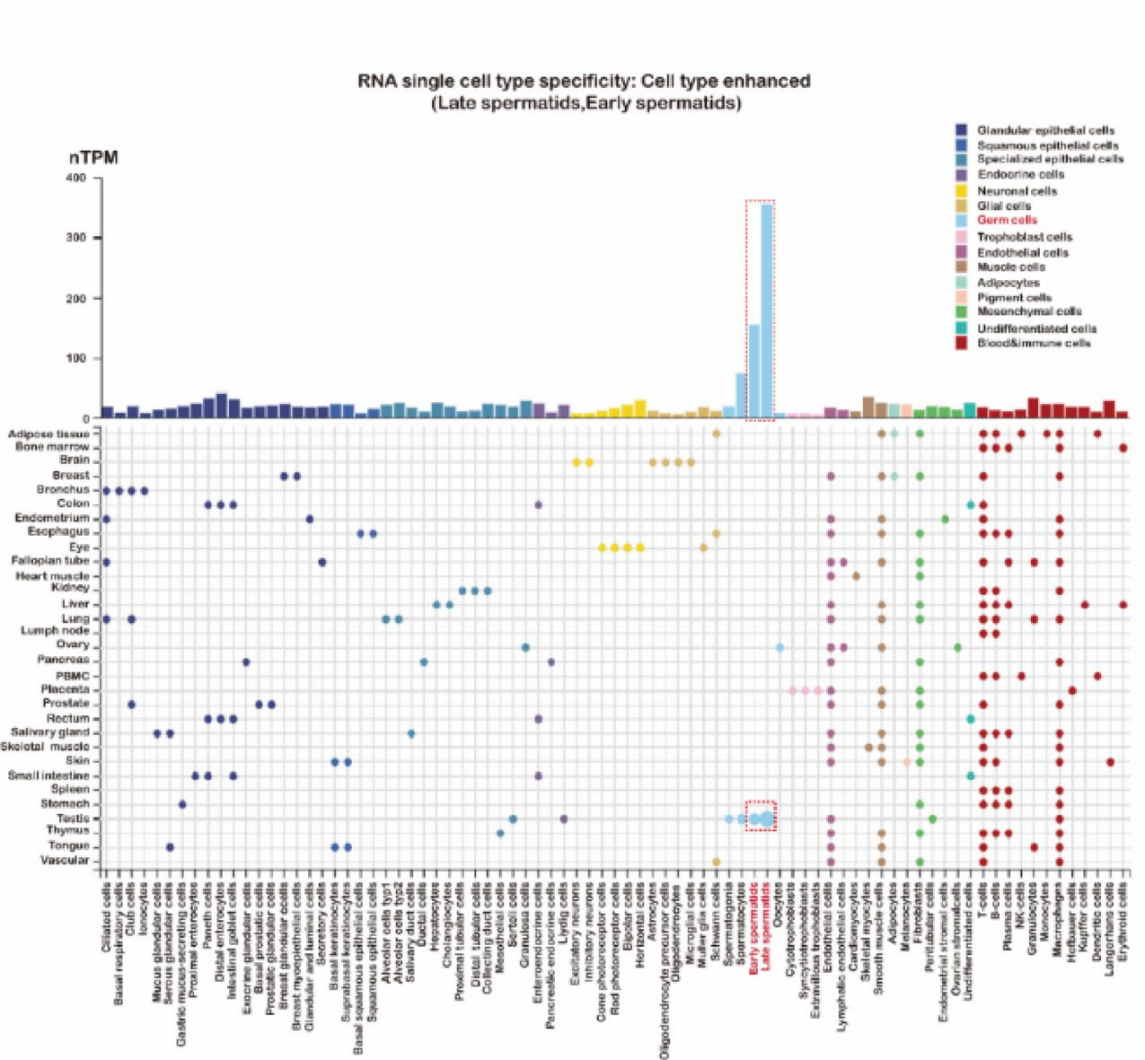
Cell type-specific expression of STK11 RNA in human tissues. Single-cell RNA sequencing analysis reveals enriched STK11 expression in germ cells, particularly in early (round) and late (elongating) spermatids.

Single-cell RNA sequencing data from the Human Protein Atlas (HPA) (https://www.proteinatlas.org/humanproteome/single+cell+type) were analyzed to evaluate STK11 expression across 31 human organs and 81 cell types. Transcripts per million (TPM) quantification revealed cell type-specific expression of STK11, with significant enrichment in germ cell lineages compared to somatic cells. Notably, expression was elevated in both early and late spermatids, peaking in late spermatids (Fig. 3, highlighted by red dashed box).

**Fig. 4:**
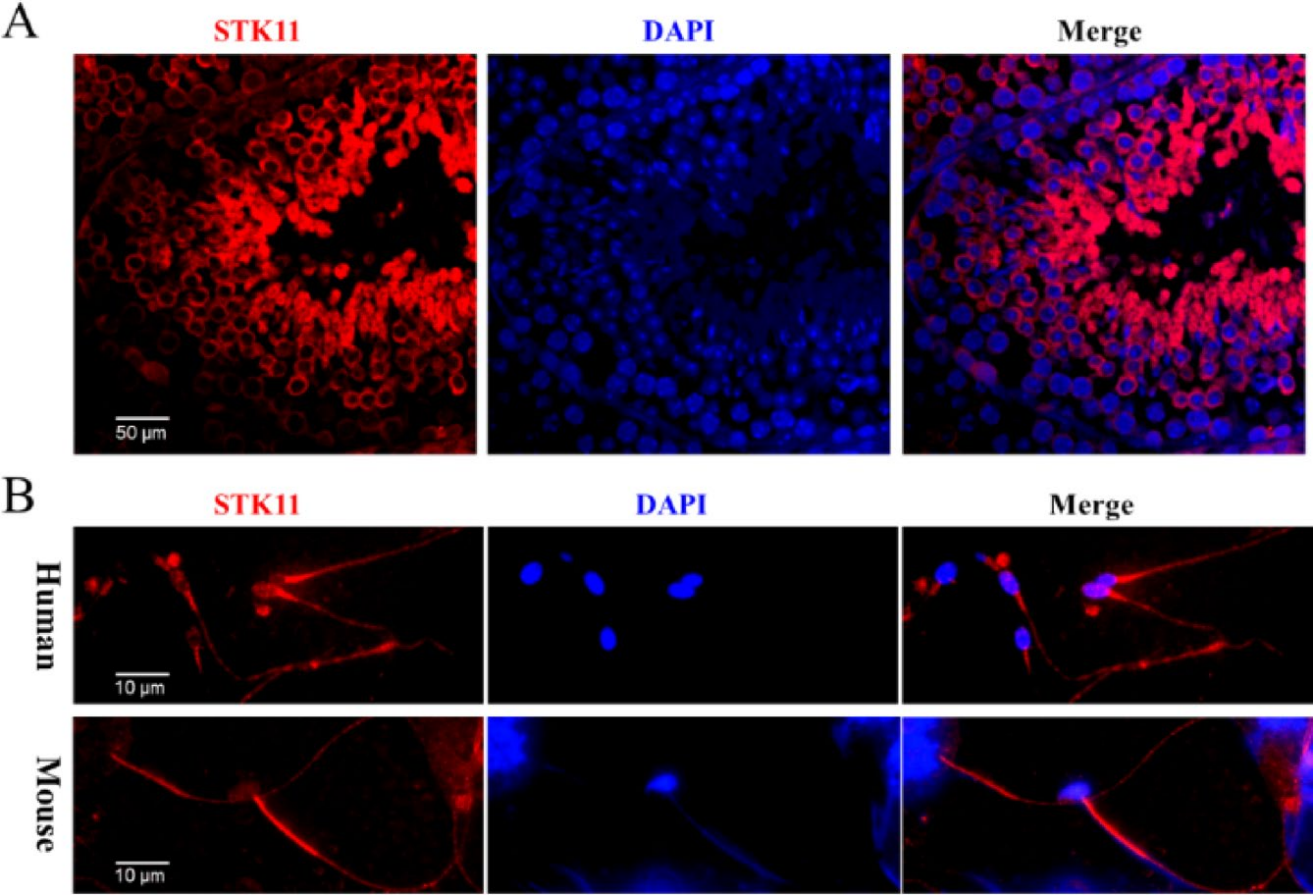
Immunolocalization of STK11 protein in the testis and mature sperm. Representative images of **(A)** mouse seminiferous tubules and **(B)** ejaculated human and mouse sperm stained with an anti-STK11 antibody (red) and DAPI (blue).

To experimentally validate these findings, we performed immunofluorescence staining for STK11 on mouse testicular sections. As shown in Fig. 4A, intense STK11 signals were detected in elongating spermatids within seminiferous tubules at stages VII–VIII, supporting a role in sperm maturation. We further examined STK11 localization in human and mouse sperm. Figure 4B shows concentrated immunoreactivity in the midpiece of the sperm tail, suggesting accumulation of STK11 in this functional compartment. This expression pattern implies a potential link between STK11 levels and sperm motility. Together, these results demonstrate that STK11 is highly expressed in testicular tissues, particularly in spermatogenic cells, with dynamic and stage-specific localization during spermatogenesis.

### *STK11* F354L mutation impairs phosphorylation of AMPK and disrupts cell polarity

**Fig. 5:**
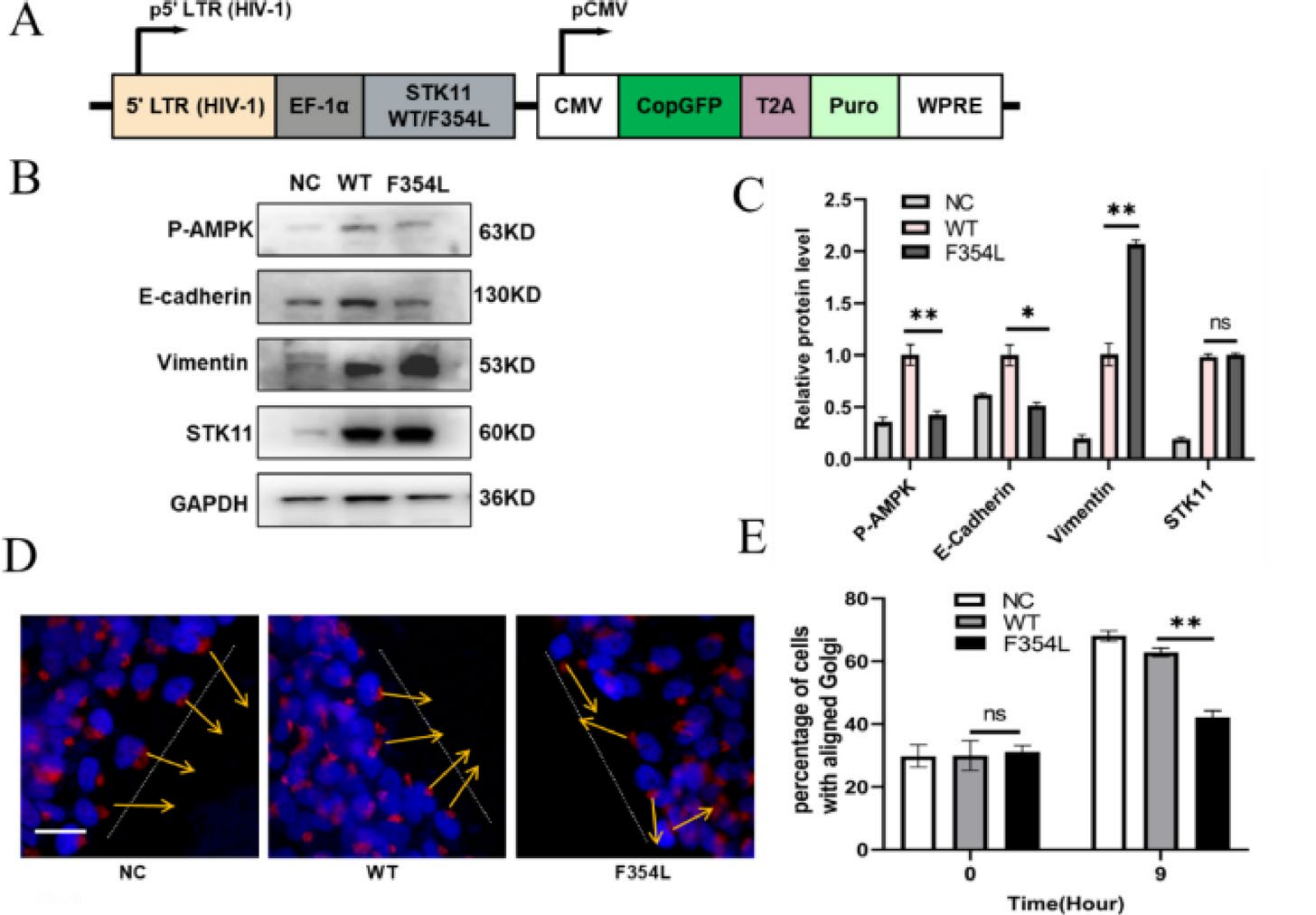
Functional analysis of *STK11*-F354L mutation in TCAM-2 cells. **(A)** The viral vectors are based on the STK11 wild-type and the F354L point mutant backgrounds, respectively. **(B)** Western blot analysis of key proteins in cells transfected with empty vector (NC), *STK11* wild-type (WT), or *STK11*-F354L mutant (F354L). Protein levels of STK11, P-AMPK, E-cadherin, and Vimentin were detected. **(C)** Quantitative Western blot results corresponding to (B). Data are presented as mean ± SD; *n* = 3. The statistical test used was an unpaired t‑test (*P < 0.05, **P < 0.01, ***P < 0.001, ****P < 0.0001, ns, no statistical significance). **(D)** Golgi reorientation assay evaluating cell polarity. TCAM-2 cells were subjected to scratch wound; Golgi apparatus (red) orientation relative to the wound direction (white dashed line) is indicated with yellow arrows. **(E)** Quantification of cells with polarized Golgi apparatus. Data are presented as mean ± SD; *n* = 400 cells per group (unpaired t‑test, *P < 0.05, **P < 0.01, ***P < 0.001, ****P < 0.0001, ns, no statistical significance).

To investigate the functional impact of the *STK11* F354L mutation, we constructed lentiviruses expressing either *STK11*-F354L or *STK11* wild-type (*STK11*-WT), along with an empty vector control. The vector backbones and their respective inserted elements are depicted in Fig. 5A. Their respective nucleotide sequence details can be found in Figure S1. Successful transduction into TCAM-2 cells was confirmed via fluorescence imaging (Fig. S2). Western blot analysis revealed that the F354L mutation significantly reduced AMPK phosphorylation and impaired AMPK pathway activation compared to *STK11*-WT (Fig. 5B).

We further observed that *STK11*-WT expression led to upregulation of E-cadherin and downregulation of Vimentin. In contrast, *STK11*-F354L resulted in opposite effects: Vimentin was elevated while E-cadherin was suppressed (Fig. 5B and C). As E-cadherin and Vimentin are established biomarkers of epithelial–mesenchymal transition (EMT)^13^, these findings suggest that the F354L mutation promotes EMT. Since loss of cell polarity is a hallmark of EMT^14,15^, these results indicate that *STK11* F354L disrupts cellular polarity and facilitates EMT progression in TCAM-2 cells.

To directly assess polarity defects, we performed a Golgi reorientation assay following scratch wound induction. The Golgi apparatus serves as a key polarization marker by reorienting toward the wound front during directed migration^16,17^. While 67% of *STK11*-WT cells exhibited properly polarized Golgi—similar to the negative control (62%)—only 41% of *STK11*-F354L-expressing cells showed correct Golgi positioning (Fig. 5D and E). This impaired reorientation indicates a definitive loss of cell polarity resulting from the F354L mutation.

## Discussion

STK11 is a serine/threonine kinase that participates in various biological processes, including cellular energy metabolism, cell cycle regulation, and cell proliferation^18^. Previous studies have indicated that mutations in the *STK11* gene are closely related to the occurrence of certain cancers^19,20^, and there have been few reports regarding its role in male spermatogenic dysfunction^21–23^. In mouse models, the deletion of STK11 has been found to be associated with decreased reproductive ability. Studies have shown that STK11-deficient mice exhibit characteristics such as reduced sperm quality and poor sperm motility^24^. This further highlights the importance of STK11 in male reproduction. Certain diseases related to mutations in the *STK11* gene may affect male fertility, such as Peutz-Jeghers syndrome^25^. Although this syndrome is primarily associated with an increased risk of tumors^26,27^, studies on related clinical cases have shown that these patients may experience difficulties in reproduction^28^.

However, the functional impact of the F354L mutation, a common mutation in the *STK11* gene^29^, remains controversial. Since the mutation site is not in the core region of the phosphorylated kinase, the effect of this amino acid change on male spermatogenesis remains uncertain^30^. Our data demonstrated that there is an association between the F354L mutation and impaired spermatogenesis and thus provides new insights into the role of the mutation in spermatogenesis. As a protein kinase, the structure of STK11 is crucial for its catalytic activity and interaction with other proteins. Any change in the structure may potentially disrupt its normal function^31^.

The F354L mutation was relatively high in our data, with a frequency of 3.52% even in the control group. The mutation frequency in East Asian populations in the database of dbGaP and BioProjectis approximately 1.8868% and 4.96%, respectively (https://www.ncbi.nlm.nih.gov/bioproject/PRJEB6930). The mutation frequency in one of these two databases was even higher than that in the control group. This difference may be due to population variation and the limited sample size. Our findings suggest that the F354L mutation has a correlation with male spermatogenic dysfunction. Our study revealed a significantly higher prevalence of the F354L mutation in infertile men with spermatogenic failure (8.33%) compared to fertile controls (3.52%), supporting a potential role in impaired spermatogenesis. Although the difference was statistically marginal (*p* = 0.043), likely due to sample size limitations, the trend is consistent with the mutation contributing to reproductive dysfunction.

To further investigate the mechanism by which the F354L mutation influences male spermatogenic dysfunction, we initially extracted data from HPA platform, which comprises 31 normal human organs and 81 annotated cell types with expression quantitation measured in TPM (Fig. 3). scRNA-seq demonstrated cell type-specific expression of STK11, with significant enrichment in germ cell lineages. Its’ expression was progressively elevated throughout spermatid development, peaking notably in late spermatids. This suggests that STK11 may play a crucial role throughout the entire process of spermatogenesis and could have a specific regulatory function in sperm function. Subsequently, we performed immunohistochemical staining on mouse testicular sections using an STK11 antibody. The staining results demonstrated that STK11 expression initiates in round spermatids and subsequently increases in elongating spermatids (Fig. 4A). This is consistent with the data from HPA. Furthermore, we performed immunofluorescence staining on both human and mouse sperm again using the STK11 antibody. The results revealed intense staining in the midpiece of the sperm tail in both species (Fig. 4B). The sperm midpiece is encapsulated by a mitochondrial sheath (comprising 70–80 helical gyres). This region is densely packed with mitochondria that power sperm motility through ATP production, which regulates the beating of the sperm flagellum. Moreover, studies have demonstrated that midpiece length exhibits a positive correlation with sperm swimming velocity—a finding consistently observed at both intraspecific and interspecific levels^32^. These findings suggest that STK11 may play a vital role in maintaining energy homeostasis and structural integrity during sperm development and function.

STK11 functions as a negative regulator of the mTOR signaling pathway by sequentially phosphorylating AMPK^33,34^. The phosphorylation and activation of AMPK by STK11 offer key mechanistic insights into its biological role^35–37^. Under metabolic stress conditions that elevate the intracellular AMP/ATP ratio, AMPK inhibits anabolic processes and promotes catabolic pathways through phosphorylation^38^. Consequently, STK11 acts as a cellular energy sensor under low-energy conditions, conferring protection against apoptosis via activation of the AMPK signaling cascade^37^. As can be seen from our Western blot data (Fig. 5B and C), the F354L mutation reduced AMPK phosphorylation and impaired AMPK pathway activation. This outcome could lead cells to promote anabolic pathways while suppressing catabolic pathways, thereby disrupting sperm energy provision, leading to diminished motility and impaired male fertility.

It has been reported that Loss of STK11 increases motility and invasiveness of breast cancer cells and induces expressions of several mesenchymal markers and an E-cadherin^39^. Li J et al. observed the localization of endogenous STK11 at the cell-cell junctions of MCF-10 A cells, where it was found to primarily co-localize with E-cadherin in adherens junctions. A significant correlation was detected between STK11 intensity and E-cadherin in TCAM-2 cells (Fig. 5B and C). Our results showed that vimentin was up-regulated while E-cadherin was down-regulated in *STK11* F354L mutation cells, whereas *STK11* wild-type induced up-regulation of E-cadherin and significant down-regulation of vimentin (Fig. 5B). This indicates that the *STK11* F354L mutation affects the epithelial-mesenchymal transition process. EMT is a biological process through which epithelial cells lose their cell polarity^40^, As STK11 is a master regulator needed for formation of proper epithelial architecture and cell polarity in mammals and participates in EMT^41,42^, our study demonstrates that the *STK11* F354L mutation impairs the regulatory role of STK11 in cell polarity.

In our study, Golgi reorientation polarity assays further demonstrated that the *STK11* F354L mutation impairs cellular polarity, leading to altered Golgi apparatus positioning. However, the molecular mechanisms through which the F354L mutation affects spermatid polarity remain unclear and warrant further in-depth investigation. Based on these findings, we speculate that males carrying the *STK11* F354L point mutation may exhibit disrupted spermatid polarity, impaired sperm motility, and consequently, defects during spermatogenesis.

In conclusion, our study provides clinical and mechanistic evidence linking the STK11 F354L mutation to male spermatogenic dysfunction. The mutation is associated with altered AMPK pathway activity, disruption of cell polarity, and potentially impaired sperm motility. However, the exact mechanisms by which polarity dysregulation affects spermatogenesis remain to be fully elucidated. Future studies using animal models carrying the F354L point mutation or expanded clinical cohorts will be essential to validate these findings and clarify the molecular pathways involved.

## Electronic Supplementary Material

Below is the link to the electronic supplementary material.

Supplementary Material 1

## Data Availability

The datasets analyzed during the current study are available in the Human Protein Atlas (HPA) repository([https://www.proteinatlas.org/humanproteome/single+cell+type]). Data are available from corresponding author upon reasonable request.

## Abbreviations

FSH: Follicle stimulating hormone
LH: Luteinizing hormone
PR: sperm progressive motility rate
T: testosterone
