# Supplementary material for: The STK11 gene F354L mutation is associated with male spermatogenic dysfunction
Source: Sci Rep. 2026 May 25;16:23817. doi: 10.1038/s41598-026-53888-4 (PMC13434688; doi:10.1038/s41598-026-53888-4)
Supplement: Supplementary file 1 [file 41598_2026_53888_MOESM1_ESM.docx]

STK11 WT 5'-3':

ATGGAGGTGGTGGACCCGCAGCAGCTGGGCATGTTCACGGAGGGCGAGCTGATGTCGGTGGGTATGGACACGTTCATCCACCGCATCGACTCCACCGAGGTCATCTACCAGCCGCGCCGCAAGCGGGCCAAGCTCATCGGCAAGTACCTGATGGGGGACCTGCTGGGGGAAGGCTCTTACGGCAAGGTGAAGGAGGTGCTGGACTCGGAGACGCTGTGCAGGAGGGCCGTCAAGATCCTCAAGAAGAAGAAGTTGCGAAGGATCCCCAACGGGGAGGCCAACGTGAAGAAGGAAATTCAACTACTGAGGAGGTTACGGCACAAAAATGTCATCCAGCTGGTGGATGTGTTATACAACGAAGAGAAGCAGAAAATGTATATGGTGATGGAGTACTGCGTGTGTGGCATGCAGGAAATGCTGGACAGCGTGCCGGAGAAGCGTTTCCCAGTGTGCCAGGCCCACGGGTACTTCTGTCAGCTGATTGACGGCCTGGAGTACCTGCATAGCCAGGGCATTGTGCACAAGGACATCAAGCCGGGGAACCTGCTGCTCACCACCGGTGGCACCCTCAAAATCTCCGACCTGGGCGTGGCCGAGGCACTGCACCCGTTCGCGGCGGACGACACCTGCCGGACCAGCCAGGGCTCCCCGGCTTTCCAGCCGCCCGAGATTGCCAACGGCCTGGACACCTTCTCCGGCTTCAAGGTGGACATCTGGTCGGCTGGGGTCACCCTCTACAACATCACCACGGGTCTGTACCCCTTCGAAGGGGACAACATCTACAAGTTGTTTGAGAACATCGGGAAGGGGAGCTACGCCATCCCGGGCGACTGTGGCCCCCCGCTCTCTGACCTGCTGAAAGGGATGCTTGAGTACGAACCGGCCAAGAGGTTCTCCATCCGGCAGATCCGGCAGCACAGCTGGTTCCGGAAGAAACATCCTCCGGCTGAAGCACCAGTGCCCATCCCACCGAGCCCAGACACCAAGGACCGGTGGCGCAGCATGACTGTGGTGCCGTACTTGGAGGACCTGCACGGCGCGGACGAGGACGAGGACCTCTTCGACATCGAGGATGACATCATCTACACTCAGGACTTCACGGTGCCCGGACAGGTCCCAGAAGAGGAGGCCAGTCACAATGGACAGCGCCGGGGCCTCCCCAAGGCCGTGTGTATGAACGGCACAGAGGCGGCGCAGCTGAGCACCAAATCCAGGGCGGAGGGCCGGGCCCCCAACCCTGCCCGCAAGGCCTGCTCCGCCAGCAGCAAGATCCGCCGGCTGTCGGCCTGCAAGCAGCAGTGA

STK11-MUT-F354L 5'-3':

ATGGAGGTGGTGGACCCGCAGCAGCTGGGCATGTTCACGGAGGGCGAGCTGATGTCGGTGGGTATGGACACGTTCATCCACCGCATCGACTCCACCGAGGTCATCTACCAGCCGCGCCGCAAGCGGGCCAAGCTCATCGGCAAGTACCTGATGGGGGACCTGCTGGGGGAAGGCTCTTACGGCAAGGTGAAGGAGGTGCTGGACTCGGAGACGCTGTGCAGGAGGGCCGTCAAGATCCTCAAGAAGAAGAAGTTGCGAAGGATCCCCAACGGGGAGGCCAACGTGAAGAAGGAAATTCAACTACTGAGGAGGTTACGGCACAAAAATGTCATCCAGCTGGTGGATGTGTTATACAACGAAGAGAAGCAGAAAATGTATATGGTGATGGAGTACTGCGTGTGTGGCATGCAGGAAATGCTGGACAGCGTGCCGGAGAAGCGTTTCCCAGTGTGCCAGGCCCACGGGTACTTCTGTCAGCTGATTGACGGCCTGGAGTACCTGCATAGCCAGGGCATTGTGCACAAGGACATCAAGCCGGGGAACCTGCTGCTCACCACCGGTGGCACCCTCAAAATCTCCGACCTGGGCGTGGCCGAGGCACTGCACCCGTTCGCGGCGGACGACACCTGCCGGACCAGCCAGGGCTCCCCGGCTTTCCAGCCGCCCGAGATTGCCAACGGCCTGGACACCTTCTCCGGCTTCAAGGTGGACATCTGGTCGGCTGGGGTCACCCTCTACAACATCACCACGGGTCTGTACCCCTTCGAAGGGGACAACATCTACAAGTTGTTTGAGAACATCGGGAAGGGGAGCTACGCCATCCCGGGCGACTGTGGCCCCCCGCTCTCTGACCTGCTGAAAGGGATGCTTGAGTACGAACCGGCCAAGAGGTTCTCCATCCGGCAGATCCGGCAGCACAGCTGGTTCCGGAAGAAACATCCTCCGGCTGAAGCACCAGTGCCCATCCCACCGAGCCCAGACACCAAGGACCGGTGGCGCAGCATGACTGTGGTGCCGTACTTGGAGGACCTGCACGGCGCGGACGAGGACGAGGACCTCTTgGACATCGAGGATGACATCATCTACACTCAGGACTTCACGGTGCCCGGACAGGTCCCAGAAGAGGAGGCCAGTCACAATGGACAGCGCCGGGGCCTCCCCAAGGCCGTGTGTATGAACGGCACAGAGGCGGCGCAGCTGAGCACCAAATCCAGGGCGGAGGGCCGGGCCCCCAACCCTGCCCGCAAGGCCTGCTCCGCCAGCAGCAAGATCCGCCGGCTGTCGGCCTGCAAGCAGCAGTGA

S1: Nucleotide sequences inserted into the lentiviral vector for STK11 wild-type and the F354L mutant.

|  |  |  |
| --- | --- | --- |
| **NC** | **WT** | **F354L** |

S2: Lentiviral transduction of TCAM-2 cells (Fluorescence imaging).
